# Clinical efficacy of azacytidine and venetoclax and prognostic impact of Tim-3 and galectin-9 in acute myeloid leukemia and high-risk myelodysplastic syndromes: A single-center real-life experience

**DOI:** 10.3389/fphar.2022.1052060

**Published:** 2022-12-21

**Authors:** Valentina Giudice, Bianca Serio, Idalucia Ferrara, Paola Manzo, Marisa Gorrese, Rita Pepe, Angela Bertolini, Francesca D’Alto, Francesco Verdesca, Maddalena Langella, Amelia Filippelli, Carmine Selleri

**Affiliations:** ^1^ Hematology and Transplant Center, University Hospital “San Giovanni di Dio e Ruggi d’Aragona”, Salerno, Italy; ^2^ Department of Medicine, University of Salerno, Baronissi, Italy; ^3^ Pharmacology Unit, University Hospital “San Giovanni di Dio e Ruggi d’Aragona”, Salerno, Italy

**Keywords:** hypomethylating agents, bcl-2 inhibitor, acute myeloid leukemia, myelodysplastic syndromes, prognosis

## Abstract

Treatment of acute myeloid leukemia (AML) and high-risk myelodysplastic syndromes (MDS) is difficult in older patients with comorbidities and high-risk disease factors. Venetoclax, the first-in-class Bcl-2 inhibitor, has proven efficacy and safety in combination with azacytidine for treatment of high-risk myeloid diseases. In this single-center real-life retrospective study, a total of 27 consecutive patients treated with azacytidine plus venetoclax were included, and clinical outcomes, hematological improvements, and biomarkers of responsiveness to therapy were compared to those observed in an historical cohort of 95 consecutive patients treated with azacytidine as single agent. Azacytidine plus venetoclax was effective and safe in older and frail AML and high-risk MDS patients, with median overall survival of 22.3 months, higher than that reported in phase III trial (14.7 months), and higher than that of historical cohort (5.94 months). Progression-free survival was higher in patients treated with the drug combination compared to those treated with azacytidine as single agent (*p* = 0.0065). Clinical benefits might increase when azacytidine and venetoclax are administered as upfront therapy (*p* = 0.0500). We showed that Tim-3 expression could be a promising therapeutic target in refractory/relapsed patients, and galectin-9 a biomarker of responsiveness to therapy. Moreover, patients treated with azacytidine and venetoclax displayed a higher overall survival regardless the presence of negative prognostic markers at diagnosis (e.g., increased *WT1* copies and/or normalized blast count). These encouraging results in a real-world setting supported efficacy and safety of azacytidine plus venetoclax as upfront therapy in AML and high-risk MDS, with clinical outcomes comparable to those of clinical trials when an appropriate venetoclax management with bone marrow assessment at every first, second, fourth, and eighth cycle, and dose adjustments for toxicities are performed.

## 1 Introduction

Acute Myeloid Leukemia (AML), a heterogeneous group of clonal hematologic malignancies, is characterized by differentiation block and increased proliferation of neoplastic clones with myeloid phenotype harboring various cytogenetic and molecular alterations (Giudice V et, 2021). Diagnosis is based on identification of at least 20% myeloblasts in the bone marrow (BM) or peripheral blood (PB) according to 2016 World Health Organization (WHO) guidelines, except for AML with specific cytogenetic abnormalities or nucleophosmin 1 (*NPM1*) mutated leukemias (Giudice V et, 2021; Heuser M et al., 2020; Khoury JD et al., 2022). AML risk evaluation is based on the European LeukemiaNet (ELN) guidelines defining three risk categories: favorable, intermediate, and adverse (Bataller A et al., 2022). In particular, combination of certain somatic mutations (e.g., biallelic mutation of *CEBPA, NPM1, FLT3-ITD,* or *TP53*) and chromosomal alterations (including t(8; 21), inv(16), or alterations involving chromosome 7) can predict AML prognosis (Bataller A et al., 2022; Fu W, et al., 2022). Myelodysplastic syndromes (MDS), another heterogenous group of clonal hematological disorders, are characterized by ineffective hematopoiesis, PB cytopenia(s), and increased risk of leukemic transformation (Giudice V et, 2021; Khoury JD et al., 2022; Hochman and Dezern, 2022). MDS pathophysiology is complex and multifactorial, where genomic instability, epigenetic changes, impaired balance between proliferation and apoptosis rate, and reduced immunological surveillance concur to dysplasia development (Patel BA et al., 2021; Hochman and Dezern, 2022). MDS classification is outlined in the 2016 and 2022 revised World Health Organization criteria and is based on cell morphology, percentage of BM blasts and/or sideroblasts, number of cytopenia(s), and the presence of cytogenetics/molecular abnormalities, such as del(5q) (Montalban-Bravo and Garcia-Manero, 2018; Khoury JD et al., 2022). Risk stratification is defined by the Revised International Prognostic Scoring System (IPSS-R) and includes percentage of BM blasts, hemoglobin levels, absolute neutrophil and platelet counts, and cytogenetics abnormalities (Greenberg PL et al., 2012). Despite great advances in disease prognostication, current risk stratification systems still lack inclusion of several parameters that influence clinical outcomes, such as the immunome role in AML transformation and progression, patients’ frailty, or molecular biology alterations (Della Porta MG et al., 2011; Bersanelli M et al., 2021). Therefore, identification of novel biomarkers is needed to better understand disease biology, identify novel therapeutical targets, and to improve patients’ prognostication.

Checkpoint receptors play a key role in self-tolerance and in the prevention of autoimmunity. T-cell immunoglobin mucin-3 (Tim-3, also known as hepatitis A virus cellular receptor 2), an inhibitory checkpoint receptor, is highly expressed by leukemic cells in several types of AML and high-risk MDS while not present on normal hematopoietic stem cells (HSCs), and its levels are associated with upregulation of proliferation and antiapoptotic genes mostly through galectin-9-mediated Tim-3 activation (Asayama T et al., 2017; Gonçalves Silva I et al., 2017; Kursunel and Esendagli, 2017). Tim-3 expressing leukemic cells show a higher frequency of somatic mutation occurrence compared to Tim-3 negative neoplastic clones, supporting its role in disease progression (Kikushige Y et al., 2010; Asayama T et al., 2017). Indeed, Tim-3 is a promising therapeutical target for AML and high-risk MDS treatment (Kikushige Y et al., 2010). Moreover, Tim-3 can be expressed on T lymphocytes and its activation induces T cell anergy and immunological synapse disruption (Achrya et al., 2020; Wolf Y et al., 2020). In AML, Tim-3-expressing T-cells are shut down by galectin-9 secreted by leukemic cells thus favoring their immunological escape. In MDS, circulating galectin-9 levels are associated with increased risk of leukemic transformation by protecting tumor cells from CD8^+^ T cell and Natural Killer (NK) cell-mediated cytotoxicity (Asayama T et al., 2017; Gonçalves Silva I et al., 2017; Wolf Y et al., 2020). Co-blockade of Tim-3 and another checkpoint receptors have shown an increased tumor cell clearance resulting in a higher efficacy of combinational immunotherapy for AML treatment in mouse models (Fourcade J et al., 2010; Zhoud et al., 2011). An ongoing phase I clinical trial is investigating efficacy and safety of anti-Tim-3 monoclonal antibody associated with a standard-of-care, decitabine, for MDS and AML showing promising preliminary results and paving the way for its potential use in hematologic malignancies (Borate U et al., 2019; Achrya et al., 2020). Hypomethylating agents, including azacytidine and decitabine, are a milestone in MDS and AML treatment, especially in older patients not eligible for hematopoietic stem cell transplantation (HSCT) (Serio B et al., 2022). These nucleoside derivatives interfere with DNA methyltransferases (DNMT) and reinduce the transcription of silenced genes and re-programming gene expression, as hypermethylation, gene silencing, and other epigenetic modifications, are a signature of myelodysplasia (Roulois D et al., 2015). However, clinical outcomes are not improved when these drugs are used as single agents, showing a median overall survival of 6–10 months (Giudice V et al., 2021; Taenaka R et al., 2022), while better outcomes are reported when associated with antiapoptotic protein inhibitors, such as venetoclax, a B-cell lymphoma-2 (Bcl-2) inhibitor first approved for chronic lymphocytic leukemia treatment, with a median survival of 18 months (DiNardo CD et al., 2020).

In this real-world study, we investigated efficacy and safety of azacytidine plus venetoclax for treatment of AML and MDS, and we compared clinical outcomes with the historical cohort of patients treated with azacytidine alone. Moreover, we investigated expression and prognostic role of Tim-3, galectin-9, and other immunological features alone and in combination with well-known diagnostic and prognostic biomarkers of AML and MDS patients for a better risk stratification.

## 2 Materials and methods

### 2.1 Patients

A total of 122 consecutive patients were included in this retrospective study after received a diagnosis of AML or MDS according to 2008 and 2016 revised WHO criteria (Vardiman JW et al., 2009; Arber DA et al., 2016), and chemotherapy as per international guidelines at the Hematology and Transplant Center, University Hospital “San Giovanni di Dio e Ruggi d’Aragona” of Salerno, Italy, from 2011 to June 2022. Risk stratification was calculated according to ELN or to IPSS-R for AML or MDS, respectively (Greenberg PL et al., 2012; Bataller A et al., 2022). International Working Group (IWG) consensus criteria were used to determine patients’ treatment response (Platzbecker U et al., 2019). Clinical characteristics at baseline are summarized in Table 1 for azacytidine historical cohort (N = 95) and azacytidine + venetoclax cohort (N = 27). Whole BM specimens were collected in ethylenediaminetetraacetic acid (EDTA) tubes for flow cytometry and gene expression analysis from patients after informed consent obtained in accordance with the Declaration of Helsinki and protocols approved by local Ethic Committee (Review Board prot./SCCE no. 151). Inclusion criteria were: age 18 or older; a diagnosis of MDS with uni- or multi-lineage dysplasia, MDS with isolated del(5q), MDS with excess of blast (EB, type 1 and 2), treatment-related MDS, chronic myelomonocytic leukemia (CMML), and *de novo* or post-MDS AML.

**TABLE 1:** Baseline characteristics.

	Aza-venetoclax cohort N = 27	Historical cohort N = 95
Median age, years (range)	70 (59–81)	73 (39–91)
Sex, n (%)		
Male	16 (59)	50 (53)
Female	11 (41)	45 (47)
WHO classification		
Single-lineage dysplasia	-	4 (4)
Multi-lineage dysplasia	-	12 (13)
MDS with isolated del(5q)	-	2 (2)
MDS-EB 1	2 (7.4)	22 (23)
MDS EB 2	3 (11.1)	28 (30)
Unclassifiable	-	1 (1)
CMML	-	2 (2)
AML *de novo*	12 (44.4)	24 (25)
AML post-MDS	10 (37.1)	-
IPSS-R risk, n (%)		
Very low	-	-
Low	1 (4)	14 (15)
Intermediate	-	21 (22)
High	9 (33)	26 (27)
Very high	5 (18)	3 (3)
Not evaluable	-	7 (7)
ELN risk, n (%)		
Favorable	7 (26)	-
Intermediate	4 (15)	-
Unfavorable	(1 4)	-
Not evaluable	-	24 (26)
Cytogenetic risk, n (%)		
Very good	-	-
Good	6 (22)	45 (47)
Intermediate	8 (30)	5 (5.3)
Poor	1 4)	2 (2.1)
Very poor	3 (11)	2 (2.1)
Not evaluable	9 (33)	41 (43)
Median % blasts (range)	32 (0–70)	11 (0–90)
First-line therapy		
Azacytidine, n (%)	12 (44)	75 (79)
Azacytidine + venetoclax	9 (33)	-
Lenalidomide, n (%)	1 (4)	6 (6)
Decitabine	3 (11)	-
Standard chemotherapy	2 (8)	5 (5)
Supportive	-	9 (10)
Second-line therapy	17/27	20/95
Azacytidine	-	11 (55)
Azacytidine + lenalidomide	-	2 (10)
Azacytidine + venetoclax	12 (71)	1 (5)
Decitabine	1 (6)	1 (5)
Standard chemotherapy	1 (6)	2 (10)
Venetoclax	3 (17)	-
Others	-	3 (15)
HSCT	3 (11)	6 (6)
Median Hb, g/dL (range)	9 (6–13)	9.1 (5.1–14.4)
Median ANC, cells/µl (range)	1,015 (30–20,850)	1,677 (120–21,490)
Median platelets/µl (range)	62,500 (6,000–216,000)	68,000 (6,000–982,000)

Abbreviations: WHO, world health organization; MDS, myelodysplastic syndrome; EB, excess of blast; CMML, chronic myelomonocytic leukemia; AML, acute myeloid leukemia; IPSS-R, international prognostic scoring system revised; ELN, european leukemia net; HSCT, hematopoietic stem cell transplantation; Hb, hemoglobin; ANC, absolute neutrophil count.

### 2.2 Treatments

Patients received chemotherapy as per international protocols (Heuser M et al., 2020; Fenaux P et al., 2021). Azacytidine was given subcutaneously at 75 mg/m^2^ for 7 days every 28 days, alone or in combination with lenalidomide or venetoclax at the maximal dose of 400 mg/daily, unless drug-related toxicity requiring dose reduction. Decitabine was administered intravenously at 20 mg/m^2^ for 5 days every 28 days. In the historical and azacytidine + venetoclax cohorts, other first or second line treatments before and after (as salvage therapy) azacytidine in monotherapy or in combination with venetoclax included: cytarabine (Ara-C) + daunorubicin (3 + 7 scheme; N = 2); reduced intensity idarubicin + Ara-C and etoposide (ICE; N = 1); reduced intensity mitoxantrone, etoposide, and Ara-C (MEC; N = 2); hydroxyurea (N = 2); fludarabine, Ara-C, and idarubicin (FLAI scheme) plus venetoclax (N = 1) followed by HSCT as per GIMEMA AML1718 protocol (EudraCT no. 2018-000392-33; approved by local Ethic Committee “Campania Sud” on 06/11/2020, prot.no 157); and fludarabine and Ara-C for 4 days, idarubicin for 3 days and G-CSF for 6 days (FLAG-Ida; N = 1). Five high-risk MDS patients were treated with azacytidine + venetoclax as second line therapy based on phase I-II clinical results showing efficacy of this combination in hematological malignancy (Santini V, 2019; Bazinet A et al, 2022). Safety was assessed as per the National Cancer Institute’s Common Terminology Criteria for Adverse Events version 4.0 (CTCAE v5.0).

### 2.3 Endpoints

Primary endpoint was hematologic improvement (HI) evaluated during the first four cycles of azacytidine + venetoclax and at the end of the eighth cycle, and defined as outlined in the 2018 revised IWG consensus criteria (Platzbecker U et al., 2019): 1) HI-erythroid (HI-E) with at least two consecutive hemoglobin (Hb) measurements >1.5 g/dl during a minimum of 16-week observation period, with transfusion independence (major response) or a minimal 50% reduction of red blood cells over a minimum of 16 weeks (minor response); 2) HI-platelets (HI-P) with an absolute increase of 30,000 platelets/µl in patients starting with 20,000/µl or increase from <20,000/µl to >20,000/µl and by at least 100%; and 3) HI-neutrophils (HI-N) with an increase of 100% and an absolute neutrophil count (ANC) > 500 cells/µl if pre-treatment <1,000 cells/µl.

Secondary endpoints were definition of overall survival (OS) and progression-free survival (PFS), assessment of changes in Wilms tumor 1 (*WT1*), Tim-3, galectin-9, and CD27 expression at the primary endpoint by real-time quantitative polymerase chain reaction (RT-qPCR), and immune cell perturbations by flow cytometry immunophenotyping.

### 2.4 Flow cytometry

For immunophenotyping, 50 µl of fresh heparinized whole BM was stained with antibodies listed in Supplementary Table S1 according to the manufacturers’ instructions and as previously described (Giudice V et al., 2021). Samples were acquired on a Navios EX (Beckman Coulter), equipped with blue (488 nm), green (532 nm), and red (633 nm) lasers, or DxFlex cytometer equipped with violet (405 nm), blue (488 nm), and red (638 nm) lasers. Our Laboratory follows the United Kingdom NEQAS for Leucocyte Immunophenotyping program for quality assessment, as per international guidelines (Reilly and Barnett, 2001). At least 1 × 10^6^ events were recorded. Post-acquisition analysis was carried out using Kaluza Analysis Flow Cytometry Software v2.1.1 (Beckman Coulter).

Cell populations were first identified based on linear parameters and then further characterized. Lymphocytes were studied for T, B, NK, and NKT cell markers, while monocytes for CD33, CD14, CD11b, CD56, CD13, CD36, CD64, CD15, and CD16 expression. Maturation profiling of CD33^+^CD56^−^ granulocytes was investigated by CD16 vs CD11b analysis. CD34^+^ HSCs were studied for CD19, CD117, and CD33, and lymphoid (CD19^+^), myeloid (CD117^+^ CD33^+^), and hematogones (CD19^+^CD34^−^CD45^+/−^) were identified. In the presence of CD45^dim^ blasts, leukemic cells were studied for CD19, CD20, CD34, CD56, CD5, CD117, CD33, CD16, CD11b, CD36, CD13, HLA-DR, CD64, CD4, CD5, CD7, CD14, CD10, CD15, CD11a, CD11c, CD45RA, CD45RO, CD61, CD42b, TdT, and MPO expression, also used for monitoring minimal residual disease (MRD).

### 2.5 *WT1* quantification

*WT1* expression levels were quantified by RT-qPCR after RNA extraction from BM mononuclear cells isolated by density gradient centrifugation using Lymphoprep (Axis-Shield Density Gradient Media, Oslo, Norway). RNA was obtained using QIAamp RNA Blood Mini Kit (Qiagen, Hilden, Germany) following manufacturer’s instructions, and quantified with a BioSpectrometer (Eppendorf, Hamburg, Germany). For cDNA synthesis, at least 1 µg of RNA was used for cDNA reverse transcription (Ipsogen RT Kit Qiagen), and then WT1-mRNA quantitative assessment was carried out using an ELN-certified Ipsogen *WT1* ProfilQuant Kit (Qiagen) as per manufacturer’s instructions.

### 2.6 Tim-3, galectin-9, and CD27 expression by RT-PCR

*TIM3*, *LGALS9*, and *CD27* expression levels were quantified by RT-qPCR at diagnosis and follow-up. Mononuclear cells were freshly isolated from BM samples by density gradient centrifugation using Clinical characteristics of samples used for *TIM3*, *LGALS9*, and *CD27* expression level analysis are summarized in Supplementary Table S2. Ficoll-Paque density gradient centrifugation (Cytiva, Marlborough, Massachusetts, United States) and subsequently subjected to RNA extraction using QIAamp RNA Blood Mini Kit (Qiagen) following manufacturer’s instructions. Clinical characteristics of samples used for TIM3, LGALS9, and CD27 expression level analysis are summarized in Supplementary Table S2. RNA was quantify using a Bio-Spec nano spectrophotometer (Shimadzu Biotech), cDNA was synthesized from 1 µg of total RNA per sample, and then genomic DNA removed (iScript gDNA CLR cDNA kit; Qiagen). RT-qPCR was performed in duplicate on a Light Cycler® 480 instrument (Roche Diagnostics) in optical 96-well plates using equal amounts (10 ng) of reverse-transcribed total RNA, iTaq universal SYBR Green Supermix (Biorad) and pre-validated primers (Biorad; Supplementary Table S3), as previously described (Asayama T et al., 2017). Glyceraldehyde-3-phosphate dehydrogenase (GAPDH) and β-actin (ACTB) were used as housekeeping genes. Samples were run in duplicate. For each gene, the mean Ct obtained from all samples was used as a calibrator. For gene expression analysis, the ΔΔCt method was employed by normalizing the ΔCt of the gene (Ct from sample—Ct from calibrator) to the ΔCt of housekeeping gene (Ct from sample—Ct from calibrator). Next, Normalized Relative Quantity (NRQ) values were obtained using the following formula (Eq. 1) and as previously described (Cilloni D and Saglio G, 2004; Hellemans J et al., 2007; Ruijter JM et al., 2015; Martins et al., 2016).

$$NRQ=\frac{E_{goi}^{Ct,goi}}{\sqrt[{f}]{\overset{f}{\underset{o}{\prod }}E_{{ref}_{0}}^{Ct,{ref}_{0}}}}$$ (1)

NRQ values were then employed for fold-change calculation (NRQ from sample/NRQ from healthy age- and gender-matched subjects). All NRQs from each sample were used in the following statistical analysis. Inter-run calibrators were used to correct technical run-to-run variation between samples analyzed in different runs.

### 2.7 Statistical analysis

Data were collected from a computerized database and chart review and were analyzed using Prism (v.9.4.1; GraphPad software, La Jolla, CA, US). Flow cytometry data were reported as percentage of positive cells, and the normalized blast count (NBC) was calculated as previously reported (Giudice V et al., 2021). *WT1* levels were reported as normalized *WT1* expression by normalizing *WT1* copy number to *ABL* copies (*WT1* copies/10^4^ *ABL* copies), as previously described (Cilloni D and Saglio G, 2004), and normal levels were <250 copies in BM (Cilloni D and Saglio G, 2004; Giudice V et al., 2021). Unpaired two-tailed t-tests for two group comparison or one-way analysis of variance (ANOVA) for multiple group comparison were performed. For group comparison, patients were divided according to disease and risk stratification, and type of therapies, and matched groups were compared to those subjects who received azacytidine + venetoclax. Low-risk MDS group was used for comparison of clinical outcomes with high-risk MDS and AML, and was not included in HI analysis, that considered only AML or high-risk MDS treated with azacytidine in monotherapy. Therefore, cases treated with lenalidomide as single agent were not included in that group of patients who has been compared to azacytidine + venetoclax cohort. Pearson analysis was used for correlation analysis. Normality was checked by D’Agostino and Pearson, Anderson-Darling, Shapiro-Wilk, and Kolmogorov-Smirnov tests. All variables passed at least one of the four tests used. The normal QQ plot is reported in Supplementary Figure S1. Outliers were tested with ROUT test, identifying 24 outliers in all studied variables (for a total of 545 values), and these outliers were mostly ANC and *WT1* values from AML subjects, therefore they were not remove from the analysis. Log-rank (Mantel-Cox) test was employed for survival analysis between groups. Multivariate linear regression for investigation of influence of clinical and biological parameters on PFS was performed using SPSS software (v.25; IBM https://www.ibm.com/support/pages/downloading-ibm-spss-statistics-25). A *p* < 0.05 was considered statistically significant.

## 3 Results

### 3.1 Patients’ characteristics: Azacytidine-venetoclax cohort

A total of 27 patients were included in the study and received azacytidine plus venetoclax as first or second or more line therapy for AML or high-risk MDS (Aza-venetoclax cohort). In this group, 81% of patients had a diagnosis of AML *de novo* (44%) or post-MDS (37%). IPSS-R was high or very high in the majority of patients (33% and 18%, respectively) in MDS patients, or ELN risk category was mostly favorable or intermediate (26% and 15%, respectively) (Table 1). Remaining patients (18.5%) had high-risk MDS, either with excess of blast (EB) of type 1 or 2. At diagnosis, 11 patients had pancytopenia, and 15 bicytopenia of whom four had neutropenia (<1,500 neutrophils/µl) and thrombocytopenia (<150,000 platelets/µl), nine had anemia (Hb < 10.0 g/dl) and thrombocytopenia, and two had anemia and neutropenia (Table 2). Median blood counts were: Hb levels, 9 g/dl (range, 6–13); absolute neutrophil count (ANC), 1,015 cells/µl (range, 30–20,850); and platelet count, 62,500 platelets/µl (range, 6,000–216,000) (Table 1).

**TABLE 2:** Hematologic improvements to azacytidine plus venetoclax.

UPN	Sex	Age	Diagnosis	IPSS-R/ELN	% blasts	Cytopenia(s) at entry			Post I cycle			Post II cycle			Post IV cycle			Post VIII cycle		
						Hb	Plt	ANC	Hb	Plt	ANC	Hb	Plt	ANC	Hb	Plt	ANC	Hb	Plt	ANC
1	F	69	AML	Favorable	70	X	X	X	Ma	HI	No	Ma	HI	No	Ma	HI	No			
2	F	76	AML	Favorable	30	X	X	X												
3	F	65	AML	Favorable	34	X	X	X	Mi	No	No	Ma	HI	HI						
4	F	62	MDS-EB 2	High	10	X	X	X	Ma	HI	No	Mi	No	No						
5	M	72	AML	Favorable	21	X	X	X												
6	M	59	MDS-EB 2	Very High	27		X	X	No	No	No	Ma	No	No						
7	M	70	MDS-EB 1	Very High	5	X	X	X												
8	M	79	MDS-EB 2	High	70	X	X		Mi	No	HI	Ma	No	No						
9	M	75	AML	High	65	X	X		Mi	No	No	Mi	No	HI	Mi	No	HI	Mi	No	HI
10	M	81	AML post	High	38	X	X		Ma	No	HI									
11	F	70	AML	Favorable	15	X	X		No	HI	No	Ma	HI	No	Ma	HI	HI	Mi	No	HI
12	M	80	AML post	Very High	39	X	X		Mi	No	No									
13	M	81	AML post	Low			X	X												
14	M	63	CMML	Very High	22	X	X	X	Mi	No	No									
15	M	73	MDS-EB 1	High	5	X	X	X	Ma	No	HI									
16	M	76	AML post	Very High	8	X	X	X												
17	F	68	AML	Intermediate	50	X	X		Ma	HI	No	Ma	No	No	Ma	HI	No	Mi	No	No
18	M	70	AML post	Unfavorable	63	X	X	X	Mi	No	No	No	No	No	Ma	No	No			
19	M	66	AML	Intermediate	26	X	X		Ma	No	HI									
20	F	68	AML	Favorable	30	X	X		Mi	HI	No	Ma	HI	No	Ma	HI	HI	Mi	No	HI
21	M	78	AML	Favorable	50	X		X	Ma	HI	No	Ma	HI	No	Ma	No	No	Mi	No	No
22	F	75	MDS-EB 2	High	60	X		X	Ma	No	No									
23	F	69	AML post	High	22	X	X		No	HI	No	Mi	No	No	Ma	No	No			
24	F	67	AML post	High																
25	M	61	AML	Intermediate	51		X	X	No	HI	HI									
26	F	77	AML	Intermediate	68		X	X	Ma	No	No	Ma	HI	No	Ma	No	No			
27	M	67	AML	Unfavorable	38	X	X	X	No	No	No	Ma	HI	HI	Mi	HI	No			

Abbreviations: IPSS-R, international prognostic scoring system revised; ELN, european leukemia net; Hb, hemoglobin; Plt, platelets; ANC, absolute neutrophil count; MDS, myelodysplastic syndrome; EB, excess of blast; CMML, chronic myelomonocytic leukemia; AML, acute myeloid leukemia; Ma, major response; Mi, minor response; HI, hematologic improvement.

Twelve patients (44%) received azacytidine alone as first line of therapy, nine (33%) azacytidine in combination with venetoclax, and of the remaining 23% of subjects, three of them received another hypomethylating agent (decitabine) instead of azacytidine as first line treatment (Table 1). Of those who did not receive venetoclax as upfront therapy, 13 of them were switched to azacytidine plus venetoclax as second line treatment with a median time of 8.4 months (range, 3.6–31.8 months), and three to venetoclax alone with a median time-to-venetoclax of 27.7 months (range, 6.1–32.3), while one patient was given decitabine alone and another one hydroxyurea, subsequently switched to azacytidine plus venetoclax as third line of therapy. One patient (UPN-14) who have received venetoclax alone as second line treatment was given FLAG-Ida as third line, and azacytidine plus venetoclax as fourth line of therapy, used as salvage and bridge treatment to HSCT. Seventeen patients (63%) died: one (UPN-25) enrolled in the GIMEMA AML1718 for transplant-related complications (TRM, transplant-related mortality) and another one (UPN-14) for TRM. Of the remaining subjects, 12 died for disease progression, and three for febrile neutropenia (UPN-3, UPN-6, and UPN-18) (Table 3).

**TABLE 3:** Univariate analysis.

Aza-venetoclax cohort					
PFS	Estimate	SE	95%CI	|t|	*p*-value
Intercept	57.40	17.55	15.89–98.91	3.270	0.0137
Age	−0.5012	0.2217	−1.025–0.02304	2.261	0.0583
Sex (Male)	2.063	2.586	−4.053–8.178	0.7976	0.4513
Diagnosis (MDS)	7.164	4.745	−4,056–18,38	1.510	0.1748
Diagnosis (AML post)	4.534	4.377	−5.816–14.88	1.036	0.3347
Hb	−0.6940	0.8803	−2.776–1.388	0.7884	0.4563
ANC	−0.0002	0.0004	−0.001–0.0008	0.4528	0.6644
Plt	<0.001	<0.001		0.3010	0.7722
Blasts (%)	0.1674	0.0666	0.0099–0.3249	2.514	0.0402
*WT1*	−0.0012	0.0007	−0.0029–0.0005	1.673	0.1383
IPSS-R/ELN (HIGH)	−14.07	4.801	−25.42–−2.717	2.931	0.0220
IPSS-R (VERY HIGH)	−8.994	5.709	−22.49–4.505	1.575	0.1591
IPSS-R/ELN (INT)	−8.673	3.668	−17.35–0.0004	2.365	0.0500
FIRST LINE (Azacytidine)	−14.15	3.627	−22.73–−5.575	3.902	0.0059
FIRST LINE (Other)	−13.18	3.643	-21.79–−4.567	3.618	0.0085
Time to venetoclax	0.9124	0.1638	0.5251–1.300	5.571	0.0008
Historical cohort					
PFS	Estimate	SE	95%CI	|t|	*p*-value
Intercept	16.66	22.89	−29.00–62.33	0.7279	0.4692
Age	−0.1382	0.2340	−0.6049–0.3285	0.5907	0.5566
Sex (Male)	−5.988	3.802	−13.57–1.597	1.575	0.1199
Diagnosis (Low-risk MDS)	8.485	6.094	−3.673–20.64	1.392	0.1683
Diagnosis (AML)	1.271	7.844	−14.38–16.92	0.1620	0.8717
Diagnosis (MDS-EB 2)	7.298	4.822	−2.323–16.92	1.513	0.1348
Hb	1.386	1.1180	−0.9675–3.739	1.175	0.2441
Plt	3.119e-005	1.39e-005	3.4e-006–5.9e-005	2.235	0.0287
ANC	−0.0012	0.0006	−0.0024–0.0001	1.830	0.0716
Blasts (%)	−0.1774	0.1346	−0.4460–0.0911	1.318	0.1918
Time to treatment (mo)	0.7137	0.1563	0.4018–1.026	4.565	<0.0001

Abbreviations: WHO, world health organization; MDS, myelodysplastic syndrome; EB, excess of blast; CMML, chronic myelomonocytic leukemia; AML, acute myeloid leukemia; IPSS-R, international prognostic scoring system revised; ELN, European LeukemiaNet; HSCT, hematopoietic stem cell transplantation; Hb, hemoglobin; ANC, absolute neutrophil count.

### 3.2 Patients’ characteristics: Historical azacytidine cohort

A total of 95 patients with a diagnosis of AML or MDS were included in the historical cohort. In this group, 25% of patients had a diagnosis of AML *de novo*. Of the MDS patients, most of them received a diagnosis of MDS-EB of type 1 or 2 (23% and 30%, respectively), or multi-lineage dysplasia (13% of cases), while single-lineage (4%), MDS with isolated del (5q) 2), or CMML (2%) less frequently observed (Table 1). IPSS-R was low in 15% of subjects, intermediate in 22% and high or very high in 30% of MDS patients (Table 1). At diagnosis, 22 patients had single lineage cytopenia (anemia, N = 8; neutropenia, N = 3; or thrombocytopenia, N = 11), 16 pancytopenia, and 42 bicytopenia of whom 17 and 14 had anemia and thrombocytopenia or neutropenia, respectively, 11 had neutropenia and thrombocytopenia (Table 2). Median blood counts were: Hb levels, 9.1 g/dl (range, 5.1–14.4); ANC, 1,677 cells/µl (range, 120–21,490); and platelet count, 68,000 platelets/µl (range, 6,000–982,000) (Table 1).

The majority of patients (79%) received azacytidine alone as first line of therapy, while only 6% of cases lenalidomide as single agent, and the remaining 15% of subjects received standard chemotherapy (e.g., reduced intensity ICE regimen) or supportive therapies. Twenty subjects were refractory/relapsed and were treated with a second line therapy, primarily azacytidine alone (55% of cases) or in combination with lenalidomide (10%). Other therapeutic choices were azacytidine plus venetoclax (N = 1), decitabine as single agent (N = 1), standard chemotherapy (N = 2), or supportive therapies (N = 3) (Table 1). Six subjects underwent to HSCT. Seventy-seven patients (81%) died for disease progression.

### 3.3 Hematologic response to azacytidine plus venetoclax

**FIGURE 1:**
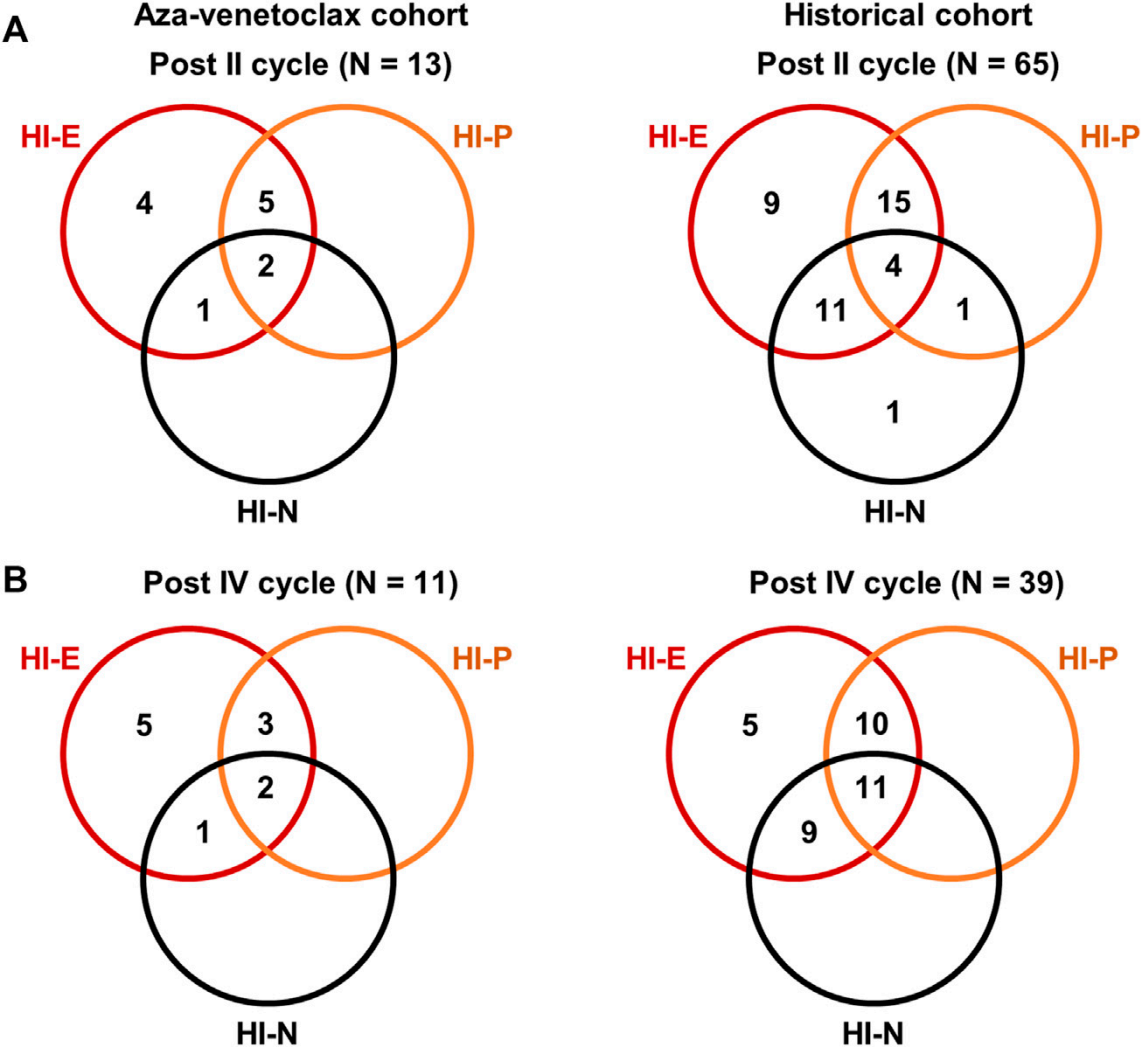
Hematological improvements (HI) of patients treated with azacytidine plus venetoclax. HI were reported for erythroid (HI-E), neutrophils (HI-N), and platelet counts (HI-P) as defined in international guidelines. Venn diagrams showing the number of patients with single lineage and multilineage responses to azacytidine plus venetoclax (aza-venetoclax cohort) or to azacytidine as single agent (historical cohort) after the second **(A)** and fourth **(B)** cycle of therapy.

In the aza-venetoclax cohort, 21 subjects were evaluable for HI assessment after the first cycle of therapy, 13 after the second administration, 11 after the fourth, and six after the eighth cycle of azacytidine plus venetoclax (Figure 1). After the first cycle, 76% of patients had a HI-E (33% minor and 43% major response), while only 38% and 24% of subjects had a HI-P and HI-N, respectively. After the second cycle, 92% of patients had a HI-E (23% minor and 69% major response), 54% HI-P, and 23% HI-N (Figure 1A). After the fourth and the eighth cycle of therapy, all evaluable patients had a HI-E (18% minor and 82% major response after the fourth cycle, and 83% minor and 17% major response after the eighth administration), while the percentage of patients who displayed a HI-P decreased after the eighth cycle from 45% to 17%. Conversely, the number of subjects with HI-N increased after the eighth cycle of azacytidine plus venetoclax from 17% to 67%.

Similarly, we evaluated HI in patients in the historical cohort after the first, second, fourth, and eighth cycle of azacytidine as single agent, and we compared the magnitude of hematological responses between cohorts. After the first cycle, a total of 83% of patients registered a HI-E (50% minor and 33% major response) that remained stable across the second and fourth cycle (53% minor and 37% major after the second cycle, and 44% minor and 46% major response after the fourth), increasing to 96% at the end of the eighth cycle (19% minor and 77% major response); however, a 4%–17% of patients never had a HI-E throughout the eight azacytidine administrations. The percentage of patients who achieved a HI-P after the first, second, fourth, and eighth cycle was 35%, 47%, 54%, and 77%, respectively, or a HI-N was 54%, 42%, 53%, and 77%, respectively (Figure 1B).

### 3.4 Clinical outcomes

**FIGURE 2:**
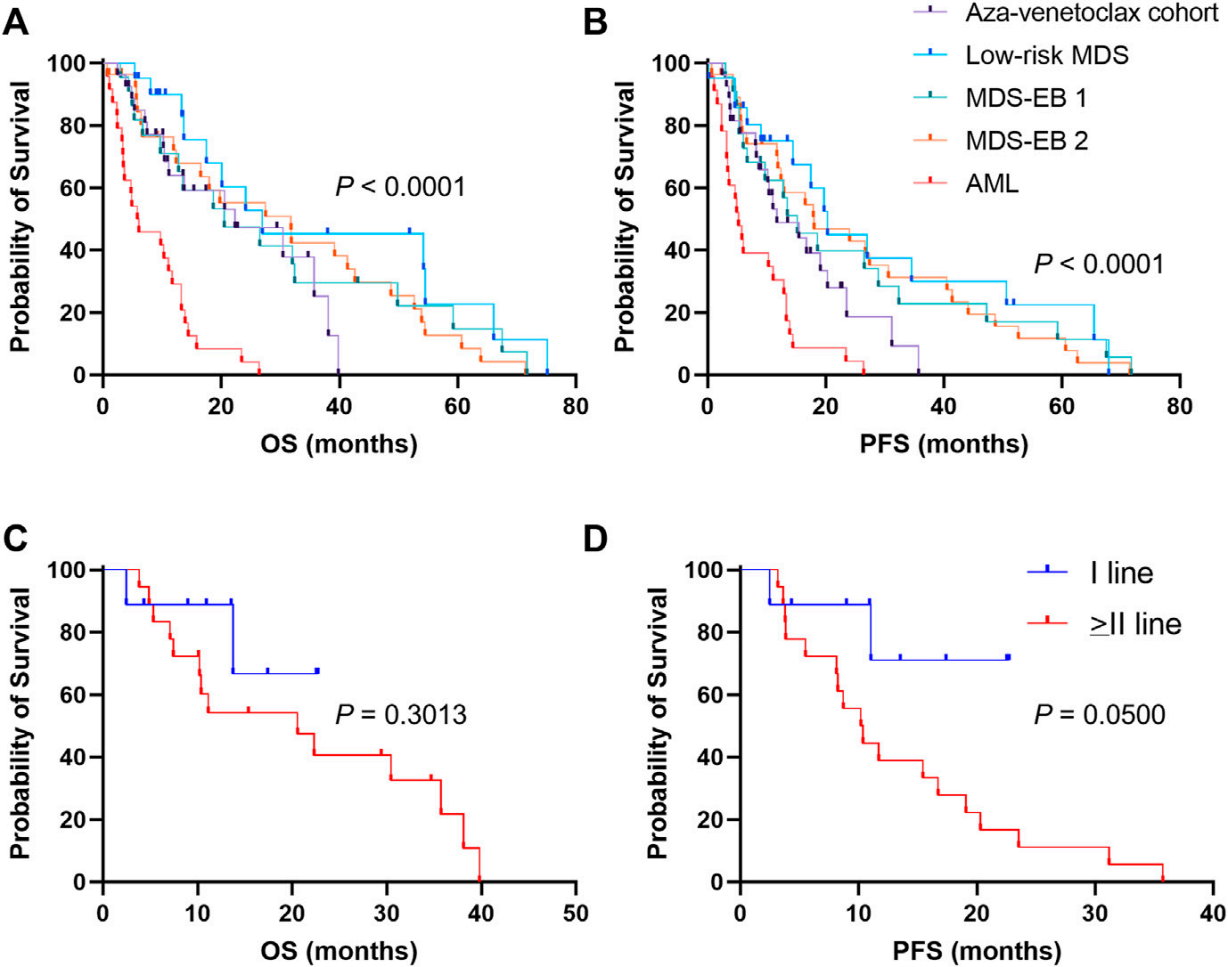
Clinical outcomes of patients treated with azacytidine plus venetoclax **(A)** overall survival (OS) and **(B)** progression-free survival (PFS) are reported for patients treated with azacytidine plus venetoclax (aza-venetoclax cohort) or to azacytidine as single agent (historical cohort), divided by diagnosis [low-risk myelodysplastic syndrome (MDS), MDS with excess of blast (EB) of type 1 and 2, and acute myeloid leukemia (AML)] **(C)** OS e **(D)** PFS are also shown for patients of the aza-venetoclax cohort treated with the drug combination as upfront therapy, or as a second or more line treatment. A *p* < 0.05 was considered statistically significant.

To investigate efficacy of azacytidine plus venetoclax in AML and high-risk MDS, OS and PFS were compared between patients treated with azacytidine as single agent (historical cohort) and those who received the combination of drugs (aza-venetoclax cohort) (Figure 2). Patients in the historical cohort were also divided based on type of diagnosis in four groups: AML, low-risk MDS, MDS-EB 1, and MDS-EB 2. Median follow-up in the historical cohort was of 12.8 months (range, 0.7 months—6.25 years), and in the aza-venetoclax group of 14 months (range, 2 months—3.3 years). Patients treated with azacytidine plus venetoclax showed a significant increase in OS compared to AML subjects treated with azacytidine as single agent [median OS, 22.3 months vs. 5.94 months, Aza-venetoclax cohort vs. Azacytidine alone in AML; *p* = 0.0003; hazard ratio (HR), 0.3025; 95% confidential interval (CI), 0.1550–0.5904] (Figure 2A). In particular, AML and high-risk MDS patients in the aza-venetoclax cohort displayed an OS similar to that of lower-/high-risk MDS treated with azacytidine as single agent (median OS, 22.3 months vs. 26.97 months vs. 20.53 months vs. 31.8 months, aza-venetoclax cohort vs. low-risk MDS vs. MDS-EB 1 vs. MDS-EB 2 treated with azacytidine as single agent; *p* = 0.3049). Similarly, patients in the azacytidine plus venetoclax cohort showed a significant higher PFS compared to AML subjects treated with azacytidine as single agent (median PFS, 11.7 months vs. 5.2 months, Aza-venetoclax cohort vs. Azacytidine alone in AML; *p* = 0.0065; HR, 0.4434; 95%CI, 0.2357–0.8342). Indeed, patients in the aza-venetoclax cohort displayed a PFS similar to that of lower-/high-risk MDS treated with azacytidine as single agent (median PFS, 11.7 months vs. 20.3 months vs. 15.2 months vs. 18 months, aza-venetoclax cohort vs. low-risk MDS vs. MDS-EB 1 vs. MDS-EB 2 treated with azacytidine as single agent; *p* = 0.2319) (Figure 2B).

We next sought to investigate clinical benefits of azacytidine plus venetoclax as frontline therapy for AML and high-risk MDS. We divided patients in three groups: azacytidine plus venetoclax as first-line (N = 9) or as second or more line of treatment (N = 18); and clinical outcomes were compared (Figures 2C, D). No significant differences were observed for OS between groups (1-year OS, 88.9% vs. 54.2%, I-line vs. > II-line; *p* = 30.13; HR, 0.4854; 95%CI, 0.1508–1.563) (Figure 2C). Conversely, patients treated with azacytidine plus venetoclax as upfront therapy displayed a slight significant higher PFS compared to those treated later with the combination, despite the small number of censored subjects per group (1-year PFS, 71.1% vs. 38.9%, I-line vs. > II-line; *p* = 0.0500; HR, 0.2702; 95% CI, 0.1030–0.7088) (Figure 2D).

### 3.5 Prognosticators

**FIGURE 3:**
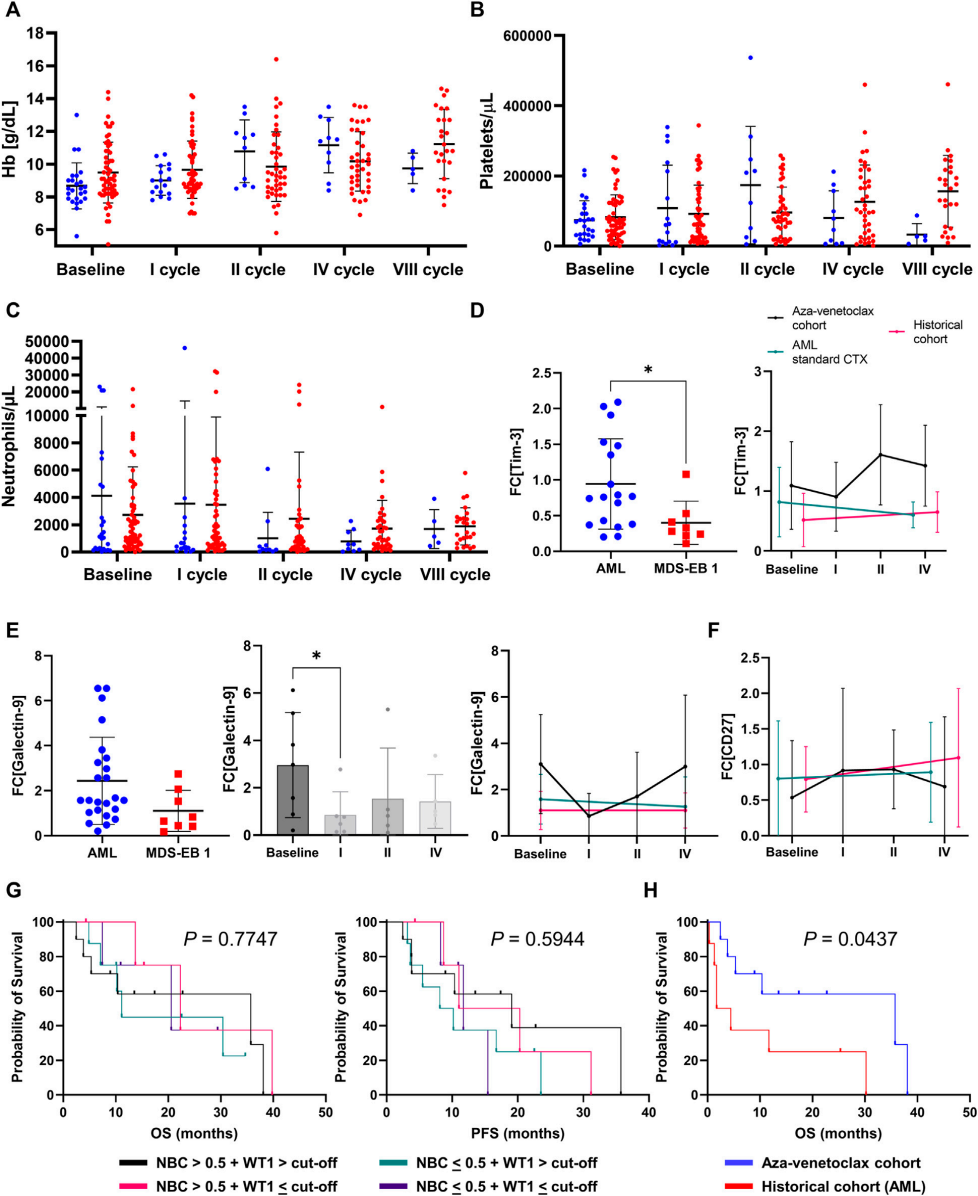
Laboratory parameters and prognosticators **(A)** hemoglobin (Hb) levels **(B)** platelets count, and **(C)** absolute neutrophil count variations at baseline and during treatment in azacytidine plus venetoclax (aza-venetoclax cohort) or to azacytidine as single agent (historical cohort) groups are reported **(D)** differences in Tim-3 expression [reported as fold-change (FC) to healthy controls] based on disease [acute myeloid leukemia (AML) and high-risk myelodysplastic syndromes (MDS) vs. MDS with excess of blast of type 1 (MDS-EB 1)] or treatment type [azacytidine + venetoclax vs. azacytidine alone vs. standard chemotherapy (CTX)]. Similarly **(E)** galectin-9 and **(F)** CD27 FC variations are displayed based on disease type and during treatment **(G)** Overall survival (OS) and progression-free survival (PFS) are reported for patients treated with azacytidine plus venetoclax divided by normalized *WT1* expression levels and flow cytometric normalized blast count (NBC) values **(H)** OS of patients with *WT1* > cut-off (50 copies in peripheral blood or 250 copies in bone marrow) + NBC >0.5 were compared between aza-venetoclax and historical AML cohort.

On univariate analysis, in aza-venetoclax cohort, a high-risk disease (*p* = 0.0220) and a first line treatment not including venetoclax (azacytidine as single agent, *p* = 0.0059; or other drugs, *p* = 0.0085) were associated with a poorer PFS, while a shorter time-to-venetoclax improved clinical outcomes of patients (*p* = 0.0008) (Table 3). In the historical cohort, normal platelet count at baseline (*p* = 0.0287) and a short time-to-treatment (*p* < 0.0001) were associated with better outcomes. Other baseline features, such as age, hemoglobin levels, or ANC, were not predictive in either aza-venetoclax or historical cohorts. In the aza-venetoclax cohort, hemoglobin levels were significantly increased already after the first cycle of therapy and reached the maximum after the fourth cycle (*p* = 0.0087 and *p* = 0.0003, respectively), while in the historical cohort, hemoglobin significantly increased only after the eighth cycle (*p* < 0.0001), as well as platelet count (*p* = 0.0048) (Figures 3A, B). No significant variations were described for ANC (Figure 3C), and between cohorts across the period of observation (all *p* > 0.05).

Next, we investigated expression and perturbations of galectin-9, Tim-3, and CD27 mRNA levels at baseline and during treatment in a subgroup of patients treated with azacytidine plus venetoclax (N = 10, at baseline, after first, second, and fourth cycle of therapy), azacytidine as single agent (N = 10), or with standard high-dose chemotherapy (N = 9) (Supplementary Table S2). At baseline, Tim-3 expression levels were significantly higher in patients with AML and high-risk MDS compared to lower-risk MDS (mean fold-change increase ± SD, 0.94 ± 0.6 vs. 0.4 ± 0.3, AML and high-risk vs. lower-risk MDS; *p* = 0.0303) (Figure 3D); however, no significant differences were described during treatment and regardless responsiveness to therapy (all *p* > 0.05). Similarly, galectin-9 mRNA expression levels tended to be higher in AML and high-risk MDS at diagnosis compared to lower risk MDS (mean fold-change increase ± SD, 2.44 ± 1.8 vs. 1.10 ± 0.9, AML and high-risk vs. lower-risk MDS; *p* = 0.0722) (Figure 3E). Galectin-9 levels significantly decreased after the first cycle of therapy (*p* = 0.0426) and tended to decrease more in those subjects who responded to azacytidine plus venetoclax (baseline vs. responders, *p* = 0.0346) compared to non-responders (responders vs. non-responders, *p* = 0.0487). No significant variations were described for CD27 expression between diseases, during treatment, or responsiveness to therapy (Figure 3F). Next, we divided patients based on normalized *WT1* expression levels and NBC values at diagnosis, as previously described (Giudice V et al, 2021), and four groups were identified: *WT1* > cut-off (250 copies for BM samples) + NBC >0.5; *WT1* > cut-off + NBC <0.5; *WT1* < cut-off + NBC >0.5; and *WT1* < cut-off + NBC <0.5. Clinical outcomes (OS and PFS) were compared between groups; however, no significant differences were observed (Figure 3G). Conversely, a significant increase in OS was described between patients with WT1 > cut-off + NBC >0.5 (the category with the highest risk) treated with azacytidine plus venetoclax and the historical AML cohort (*p* = 0.0437) (Figure 3H).

**FIGURE 4:**
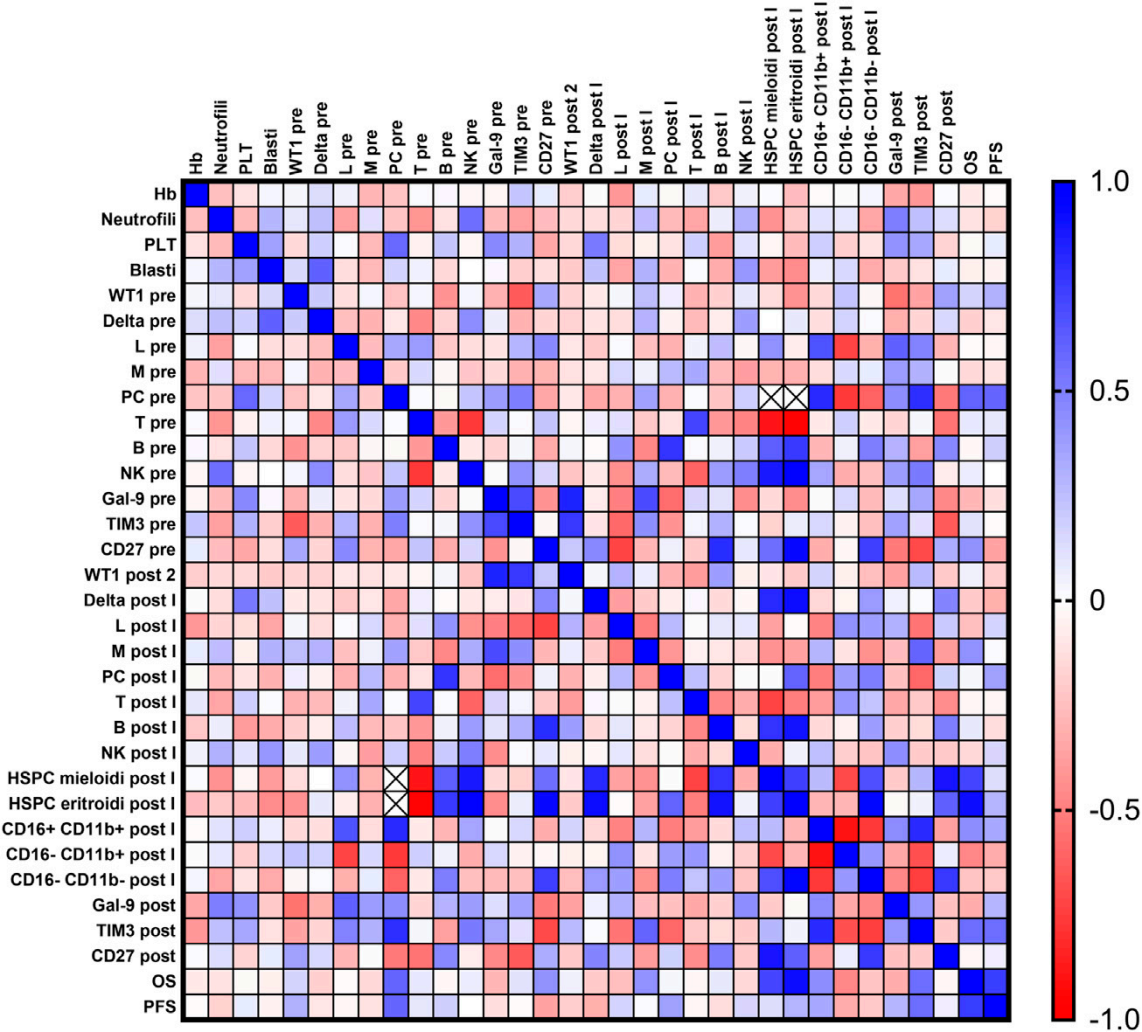
Correlation analysis. Blood counts [hemoglobin (Hb), platelet count (PLT), absolute neutrophil count (ANC)], percent of bone marrow blasts, normalized *WT1* mRNA expression, flow cytometry data [% of lymphocytes (Lymph), monocytes (Mono), plasma cells, T and B cells, Natural Killer (NK) cells, myeloid and erythroid hematopoietic stem and progenitor cells (HSPCs), and granulocyte maturation curve], galectin-9 (Gal-9), Tim-3, and CD27 expression levels, overall survival (OS), and progression-free survival (PFS) were compared at baseline and after treatment by Pearson correlation analysis. Positive correlations are displayed in blue (r = 1), and negative correlations in red (r = −1).

Pearson correlation analysis was performed between all clinical features, flow cytometry frequencies, and gene expression levels (Figure 4). ANC was inversely correlated with T lymphocyte frequency at baseline (r = −0.414; *p* = 0.0497) and positively with NK cells (r = 0.555; *p* = 0.0059), and platelet counts were correlated with plasma cells frequency at diagnosis (r = 0.585; *p* = 0.0219). Galectin-9 and Tim-3 expression levels at diagnosis were positively correlated (r = 0.703, *p* = 0.0348), and Tim-3 expression after therapy was positively correlated with mature CD16^+^CD11b + granulocytes (r = 0.805; *p* = 0.0088) and negatively correlated with intermediate CD16^−^CD11b + (r = −0.674; *p* = 0.0464) and immature CD16^−^CD11b-granulocytes post treatment (r = −0.743; *p* = 0.0219). Conversely, CD27 expression levels after therapy were positively correlated with immature granulocyte frequency (r = 0.759; *p* = 0.0177) and percentage of myeloid hematopoietic stem and progenitor cells (HSPCs) (r = 0.886, *p* = 0.0454).

Finally, a multivariate analysis was performed to identify factors that significantly influenced PFS in AML and high-risk MDS patients treated with azacytidine + venetoclax (Table 4). Age and percentage of BM blasts were significantly associated with PFS in multivariate analysis (*p* = 0.016, and *p* = 0.012, respectively), while no associations were described between other clinical and molecular features, such as *WT1* expression (*p* = 0.121) and galectin-9 or Tim-3 at baseline (*p* = 0.469 or *p* = 0.225, respectively).

**TABLE 4:** Multivariate analysis.

Aza-venetoclax cohort					
PFS	Estimate	SE	95%CI	|t|	*p*-value
Age	0.114	0.047	1.022–1.229	1.120	0.016
Sex (Male)	0.396	0.585	0.472–4.670	1.485	0.499
Blasts (%)	−0.046	0.018	0.922–0.990	0.955	0.012
IPSS-R/ELN (HIGH)	1.156	0.668	0.858–11.757	3.177	0.083
FIRST LINE (Other)	1.5	0.905	0.761–26.395	4.483	0.097

Abbreviations: IPSS-R, international prognostic scoring system revised; ELN, European LeukemiaNet.

## 4 Discussion

AML and MDS are heterogenous groups of hematologic malignancies characterized by various phenotypic and genomic abnormalities diversely influencing clinical outcomes, and mostly affecting older patients who are frequently not eligible for high-dose chemotherapy and HSCT due to comorbidities, poor performance status, and high-risk disease associated factors (Cherry EM et al., 2021; Winters AC et al., 2022). Therefore, alternative more manageable therapeutic strategies are required. Hypomethylating agents, such as azacytidine and decitabine, have become a milestone in treatment of older AML and high-risk MDS patients with good efficacy and safety profile (Serio B et al., 2022). In 2018, the Bcl-2 inhibitor venetoclax has been added to hypomethylating agents or low-dose cytarabine for treatment of newly diagnosed AML patients aged 75 or older not eligible for high-dose standard chemotherapy based on phase III clinical trial results (DiNardo CD et al., 2020; Stein EM et al., 2020; Pollyea DA et al., 2021; Labrador J et al., 2022; Mustafa Ali MK et al., 2022; Wolach O et al., 2022). However, real-world data are still few. Here, we reported a single-center real-life retrospective study on efficacy, safety, and prognosticators of AML and high-risk MDS patients treated with azacytidine plus venetoclax, and results were compared to our historical cohort of patients treated with azacytidine as single agent.

In phase III clinical trials, the combination of azacytidine plus venetoclax has shown a rate of complete remission (CR) of 36.7% and of composite CR (CCR, CR plus CR with incomplete hematologic recovery) of 66.4%, higher than that reported in historical cohorts (DiNardo CD et al., 2020). In our single-center real-life experience, CR rate was 15%–17% throughout the second to the eighth cycle of therapy, with a CCR of 54%–67% and a single-lineage HI of 31%–46% (especially HI-E) in the same observation period. The rate of non-responders was 8% after the second cycle, and then all patients achieved at least a single-lineage improvement. Of note, responsiveness to therapy was investigated on BM evaluation performed after the first, second, fourth, and eighth cycle, adding evidence to the need of serial BM evaluation for a more sensitive disease monitoring (Percival ME et al., 2017). In our historical cohort, CR rates ranged from 6% to 58%, and CCR rates from 48% to 93% after the second to the eighth cycle of therapy, according to the kinetics of azacytidine in blood count improvement after six-eight cycles of therapy (Stein EM et al., 2020). A small proportion of patients (10%) was still non-responder to azacytidine as single agent after the fourth cycle of therapy. In the subgroup of AML patients (N = 22) treated with azacytidine alone, CCR rates were 50%–53% during the first four cycle of therapy, and reached 100% (CR rate, 43%) after the eighth cycle. Our CR and CCR rates in the historical cohort were higher than those reported, likely because only 45% and 32% of AML patients in the historical cohort survived to the fourth and eight cycle, respectively, and were those subjects with robust response to therapy. Our results were similar to those reported in other real-world studies including AML patients treated with azacytidine plus venetoclax (Brancati S et al., 2021; Gozzo L et al., 2021). Indeed, in previously published AML case series (N = 21), a CR rate of 52% and a CCR of 67% has been reported, with a median duration of response of 4.5 months (range, 0.5–12.5 months). Therefore, our data confirmed efficacy of azacytidine plus venetoclax in AML and high-risk MDS by inducing a faster and durable hematological improvement already after the second cycle of therapy.

In our cohort, 37.1% and 18.5% of our patients had a diagnosis of secondary AML or high-risk MDS, in contrast to only a 25% and 43.8% of the VIALE-A phase III trial and the Vachhani’s real-life experience (DiNardo CD et al., 2020; Vachhani P et al., 2022). Moreover, we included patients previously treated with hypomethylating agents in monotherapy. Our follow-up period was similar to that in the phase III VIALE-A trial (14 months vs. 20.5 months, respectively) and higher than other real-life experiences (e.g., 7.2 months). Median OS of patients treated with azacytidine plus venetoclax was 22.3 months, higher than that reported in the VIALE-A trial (14.7 months) and in other real-life experiences (8.6 months), and higher than that of our historical group (5.94 months in AML patients treated with azacytidine as single agent) and other historical cohorts (9.6 months) (DiNardo CD et al., 2020). Indeed, our AML and high-risk MDS patients treated with azacytidine plus venetoclax showed clinical outcomes similar to those reported in lower-risk MDS patients (median OS, 22.3 months vs. 26.97 months vs. 20.53 months vs. 31.8 months, aza-venetoclax cohort vs. low-risk MDS vs. MDS-EB 1 vs. MDS-EB 2 treated with azacytidine as single agent; *p* = 0.3049). Moreover, PFS was similar between AML and high-risk MDS treated with the combination of drugs and lower-/high-risk MDS treated with azacytidine as single agent (median PFS, 11.7 months vs. 20.3 months vs. 15.2 months vs. 18 months, aza-venetoclax cohort vs. low-risk MDS vs. MDS-EB 1 vs. MDS-EB 2 treated with azacytidine as single agent; *p* = 0.2319). These encouraging results in a real-world setting could be linked with the use of azacytidine plus venetoclax as upfront therapy in high-risk diseases, as we reported a significant higher PFS compared to those treated later with the combination, despite the small number of censored subjects per group (1-year PFS, 71.1% vs. 38.9%, I-line vs. > II-line; *p* = 0.0500). On univariate analysis, a shorter time-to-venetoclax was linked to improved clinical outcomes (*p* = 0.0008). Moreover, in our cohort, 11% patients were bridged to HSCT, a rate higher than that previously reported (7.1% and 0.7% in Vachhani’s and VIALE-A studies, respectively) (DiNardo CD et al., 2020; Vachhani P et al., 2022).

In the VIALE-A study, grade 3 or higher thrombocytopenia occurred in 45% of cases, neutropenia in 42%, and febrile neutropenia in 42% (19%, grade IV) (DiNardo CD et al., 2020). In our cohort, grade 3 or higher thrombocytopenia occurred in 53%, 30%, 50%, and 80% of cases, after the first, second, fourth, and eighth cycle of therapy, while grade 3 or higher neutropenia in 65%, 80%, 67%, and 40% of cases after the first, second, fourth, and eighth cycle of treatment. Febrile neutropenia was observed in 19% of patients, and in 3 cases (11%) was of grade IV.

Finally, we also sought to investigate candidate biomarkers of responsiveness to azacytidine plus venetoclax therapy, and well-established biomarkers (e.g., *WT1* mRNA levels or NBC) or other novel molecules were studied, such as Tim-3 expression levels. For example, Tim-3, a checkpoint receptor, is a promising targeted therapy in cancer immunotherapy, and its inhibitor, cobolimab, has displayed efficacy and safety as monotherapy or in combination with other checkpoint inhibitors in patients with advanced solid tumors in phase I AMBER study (Falchook GS et al., 2022), and has been also proposed in high-risk MDS (Lee P et al., 2021). Indeed, Tim-3 expression is increased on blasts and in high-risk hematological diseases (Asayama T et al., 2017), together with its pathway companion, galectin-9, and proliferation of Tim-3^+^ MDS blasts can be inhibited by anti-Tim-3 antibody (Gonçalves Silva I et al., 2017; Wolf Y et al., 2020). We confirmed that Tim-3 expression was increased in AML and high-risk MDS compared to lower-risk MDS; however, its levels were not significantly affected by treatments and were not correlated with responsiveness to therapy, either azacytidine as single agent or in combination with venetoclax. These results might be explained by the use of mRNA from bulk BM samples for qRT-PCR, thus including both blasts and immune cells in the analysis. Venetoclax has also immune-enhancing activities by boosting T cell effector cytotoxic functions, while azacytidine induces expansion of T regulatory cells and favors T cell-mediated killing of leukemic cells (Jia X et al., 2020; Lee JB et al., 2021). Therefore, we might speculate that increased Tim-3 expression at diagnosis could be linked to high disease burden and/or to an NK cell-mediated cytotoxicity, while after treatment to an increased immune response, that might be ineffective or impaired, thus supporting the use of cobolimab as a possible salvage therapy in refractory/relapsed AML and high-risk patients. Tim-3 expression increase after the second cycle of therapy might be explained by: i) a restoration of immune responses, and ii) by the recovery of normal hemopoiesis, as Tim-3 is also present at low level on granulocyte-monocyte progenitors (Kikushige Y et al., 2010). Indeed, in solid tumors, galectin-9 inhibition rescues the transition from exhausted to terminally exhausted T cells by reducing galectin-9-induced cell death and by expanding cytotoxic CD8^+^ T cells (Yang R et al., 2021). Moreover, in rheumatoid arthritis, galectin-9 is higher in patients with severe disease, and responders display a significant decrease after treatment, both expression on lymphocytes and plasma levels (Sun J et al., 2021). Therefore, expansion of Tim-3^+^ lymphocytes after treatment might induce re-expression of galectin-9 in a positive feedback. Conversely, galectin-9 could be proposed as a candidate biomarker of responsiveness to therapy. WT1-mRNA is used as a specific and sensitive diagnostic and prognostic marker of AML and MDS, especially in the absence of specific molecular signature, as expression levels can mirror disease progression and identify MDS patients with poorer prognosis (Giudice V et al., 2021). Here, we confirmed the prognostic impact of flow cytometry parameters, such as NBC, hematogones, and erythroid HSPCs, while we showed that patients treated with azacytidine plus venetoclax had better clinical outcomes regardless the presence of negative prognostic markers at diagnosis, like increased WT1-mRNA levels and NBC.

Limitations of our study are: 1) the small number of patients in the azacytidine + venetoclax cohort, especially those evaluable at the end of the eighth cycle of therapy; 2) the small number of samples used for Tim-3, galectin-9, and CD27 expression analysis; 3) and lack results on quality of life of patients treated with standard of care or azacytdine + venetoclax.

In conclusions, in this single-center real-world experience of azacytidine plus venetoclax for AML and high-risk MDS treatment, we showed that this drug combination at suggested dosages was effective and safe in older and frail AML and high-risk MDS patients, and clinical benefits might increase when azacytidine and venetoclax are administered as upfront therapy. We also reported follow-up time and clinical outcomes similar to those of clinical trials when an appropriate venetoclax management with BM assessment at every first, second, fourth, and eighth cycle and dose adjustments for toxicities are performed. Moreover, we showed that Tim-3 expression could be a promising therapeutic target in refractory/relapsed patients, and galectin-9 a biomarker of responsiveness to therapy.

## Data Availability

The original contributions presented in the study are included in the article/Supplementary Materials, further inquiries can be directed to the corresponding author.
