# Supplementary material for: Clinical efficacy of azacytidine and venetoclax and prognostic impact of Tim-3 and galectin-9 in acute myeloid leukemia and high-risk myelodysplastic syndromes: A single-center real-life experience
Source: Front Pharmacol. 2022 Dec 21;13:1052060. doi: 10.3389/fphar.2022.1052060 (PMC9810751; doi:10.3389/fphar.2022.1052060)
Supplement: Supplementary file 1 [file DataSheet1.docx]

**Supplementary Tables**

**Supplementary Table 1. Antibodies’ specifications.**

| Antigen | Fluorochrome | Clone |
| --- | --- | --- |
| CD3 | APC | UCHT1 |
| CD4 | PE | 13B8.2 |
| CD8 | APC-A750 | B9.11 |
| CD5 | PC7 | BL1a |
| CD7 | PC7 | 8H8.1 |
| CD19 | PC5.5 | J3-119 |
| CD20 | PB | B9E9 |
| SmIg-kappa/SmIg-lambda/CD19 | FITC/PE/ECD | Polyclonal/Polyclonal/J3-119 |
| CD56 | ECD | N901 |
| CD16 | PB | 3G8 |
| CD10 | PC7 | ALB1 |
| CD11a | FITC | 25.3 |
| CD11b | PC7 | Bear1 |
| CD11c | PE | BU15 |
| CD13 | PC5.5 | Immu103.44 |
| CD14 | APC-A750 | RMO52 |
| CD15 | PE | 80H5 |
| CD33 | APC | D3HL60.251 |
| CD34 | APC700 | 581 |
| CD36 | FITC | FA6.152 |
| CD64 | ECD | 22 |
| CD117 | PE | 104D2D1 |
| HLA-DR | FITC | Immu-357 |
| CD45 | KO | J33 |
| CD45RA | FITC | ALB11 |
| CD45R0 | ECD | UCHL1 |
| CD71 | FITC | YDJ1.2.2 |
| CD61 | FITC | SZ21 |
| CD42b | PE | SZ2 |
| TdT | FITC | HT1+HT4+HT8+HT9 |
| MPO | PE | CLB-MPO-1 |

Abbreviations: CD, cluster of differentiation; TdT, terminal deoxynucleotidyl transferase; MPO, myeloperoxidase; APC, allophycocyanin; PE, phycoerythrin; PC, phycoerythrin cyanin; PB, pacific blue; FITC, fluorescein isothiocyanate; ECD, PE-Texas Red; KO, krome orange.

**Supplementary Table 2. Clinical characteristics of patients studied for TIM3, galectin-9, and CD27 expression levels.**

|  | Azacytidine + venetoclax  N = 10 | Standard chemoterapy  N = 9 | Azacytidine in monotherapy  N = 10 |
| --- | --- | --- | --- |
| Mean age, years (range) | 72 (62-78) | 61 (29-75) | 70 (57-83) |
| M/F | 3/7 | 6/3 | 7/3 |
| Diagnosis  AML  High-risk MDS  Low-risk MDS  CMML | 6  4  -  - | 9  -  -  - | -  2  6  2 |
| Mean Hb, g/dL (range= | 9 (8-11) | 10.3 (8.7-13) | 10.3 (8.5-13.4) |
| Mean PLTx10^3^/µL (range) | 109 (31-216) | 93 (11-259) | 208 (24-963) |
| Mean ANC, cells/µL (range) | 1366 (30-4788) | 4415 (809-16079) | 1892 (160-7366) |
| WT1 copy number (range) | 1712 (66-6464) | 9708 (25-34537) | 2084 (1-6652) |
| NBC (range) | 13 (0.012-60) | 4.18 (0.07-29.7) | 0.28 (0.13-0.86) |

**Abbreviations.** AML, acute myeloid leukemia; MDS, myelodysplastic syndromes; CMML, chronic myelomonocytic leukemia; Hb, hemoglobin; PLT, platelet count; ANC, absolute neutrophil count; WT1, Wilms’ tumor 1; NBC, normalized blast count.

**Supplementary Table 3. Primers.**

| Gene symbol | Unique Assay ID | Gene name | Entrez Gene ID | Amplicon size |
| --- | --- | --- | --- | --- |
| TIM3 | qHsaCED0044589 | **Hepatitis A virus cellular receptor 2** | 84868 | 63 |
| LGALS9 | qHsaCID0014464 | **Galectin-9** | 3965 | 177 |
| CD27 | qHsaCID0017180 | **CD27 molecule** | 939 | 109 |
| ACTB | qHsaCED0036269 | **β-actin** | 60 | 62 |
| GAPDH | qHsaCED0038674 | **glyceraldehyde-3-phosphate dehydrogenase** | 2597 | 117 |

**Supplementary Figure 1.** Normal QQ plot for normality test.
